# Association of a low vitamin D status with risk of post-stroke depression: A meta-analysis and systematic review

**DOI:** 10.3389/fnut.2023.1142035

**Published:** 2023-02-16

**Authors:** Kuo-Chuan Hung, Jheng-Yan Wu, Amina M. Illias, Chong-Chi Chiu, Ying-Jen Chang, Shu-Wei Liao, Kuei-Fen Wang, I-Wen Chen, Cheuk-Kwan Sun

**Affiliations:** ^1^Department of Anesthesiology, Chi Mei Medical Center, Tainan City, Taiwan; ^2^School of Medicine, College of Medicine, National Sun Yat-sen University, Kaohsiung City, Taiwan; ^3^Department of Nutrition, Chi Mei Medical Center, Tainan City, Taiwan; ^4^Department of Anesthesiology, Chang Gung Memorial Hospital, Taoyuan City, Taiwan; ^5^Graduate Institute of Clinical Medical Sciences, College of Medicine, Chang Gung University, Taoyuan City, Taiwan; ^6^Department of General Surgery, E-Da Cancer Hospital, Kaohsiung City, Taiwan; ^7^School of Medicine, College of Medicine, I-Shou University, Kaohsiung City, Taiwan; ^8^Department of Medical Education and Research, E-Da Cancer Hospital, Kaohsiung City, Taiwan; ^9^Department of Anesthesiology, Chi Mei Medical Center, Liouying, Tainan City, Taiwan; ^10^Department of Emergency Medicine, E-Da Hospital, I-Shou University, Kaohsiung City, Taiwan

**Keywords:** vitamin D, stroke, depression, nutrition, post-stroke depression

## Abstract

**Background:**

Although post-stroke depression (PSD) affects one-third of patients following an acute stroke, pooled evidence addressing the correlation between a low vitamin D status and the risk of PSD remains inconclusive.

**Methods:**

Comprehensive database search of Medline, EMBASE, Cochrane library, and Google Scholar was performed from inception to December 2022. The primary outcome was the association of PSD risk with a low vitamin D status, while the secondary outcomes included the relationship between PSD and other risk factors.

**Results:**

Analysis of seven observational studies published between 2014 and 2022 with 1,580 patients showed pooled incidences of vitamin D deficiency (defined as 25[OH] D levels < 50 nmol/L) and PSD of 60.1 and 26.1%, respectively. Patients with PSD had a lower circulating vitamin D concentration compared to those without [mean difference (MD) =−13.94 nmol/L, 95% CI: −21.83 to −6.05, *p* = 0.0005, *I*^2^ = 91%, six studies, 1,414 patients]. Meta-analysis also demonstrated a correlation between a low vitamin D level and an increased PSD risk [odd ratio (OR) = 3.25, 95% CI: 1.57–6.69, *p* = 0.001, *I*^2^ = 78.7%, 1,108 patients], the heterogeneity of which was found to be associated with the incidence of vitamin D deficiency but not female proportion on meta-regression. Besides, female gender (OR = 1.78, 95% CI: 1.3–2.44, *p* = 0.003, *I*^2^ = 31%, five studies, 1,220 patients), hyperlipidemia (OR = 1.55, 95% CI: 1.01–2.36, *p* = 0.04, *I*^2^ = 0%, four studies, 976 patients), and high National Institutes of Health Stroke Scale (NIHSS) scores (MD = 1.45, 95% CI: 0.58–2.32, *p* = 0.001, *I*^2^ = 82%, five studies, 1,220 patients) were potential risk factors for PSD. For the primary outcome, the certainty of evidence was very low. Regarding secondary outcomes, the certainty of evidence was low for BMI, female gender, hypertension, diabetes, and stroke history, and very low for age, level of education, hyperlipidemia, cardiovascular disease, and NIHSS scores.

**Conclusion:**

The results suggested an association of a low circulating vitamin D level with an increased risk of PSD. Besides, female gender, hyperlipidemia, high NIHSS score were related to an increased risk or occurrence of PSD. The current study may imply the necessity of routine circulating vitamin D screening in this population.

**Systematic review registration:**

https://www.crd.york.ac.uk/prospero/, identifier CRD42022381580.

## 1. Introduction

Post-stroke depression (PSD), which is frequently observed weeks and months following an acute stroke, is a common neuropsychiatric sequela (1, 2). In addition to psychosocial factors, the etiology of PSD is multifactorial involving a myriad of pathophysiological changes including dysregulation of the hypothalamic-pituitary-adrenal (HPA) axis, abnormal neurotrophic response, reduced levels of monoamines, glutamate-mediated excitotoxicity, and increased inflammation (3, 4). Prior investigations have hypothesized that focal brain injury-induced hyperactivation of the HPA axis could cause an increased production of HPA hormones, thereby changing the expressions of corticoid receptors primarily in the limbic system. The resulting alterations in the negative feedback mechanism then contribute to both morphological and functional impairments of key brain areas with a high density of glucocorticoid receptors, in particular the hippocampus (5), which is pivotal in governing memory and emotions. The damage may predispose to the development of cognitive and depressive disorders (6). Inflammation in the brain has also been reported to play a role in the occurrence of depression (7). Pro-inflammatory cytokines have been found to affect the concentrations of serotonin (5-hydroxytryptamine, 5-HT) and norepinephrine, thereby altering transmission in the neural circuits (8). Moreover, inflammation is known to activate the response system to stress (9). In concert with this finding, compared with individuals without depression, the stress response of those with depression has been shown to be over-activated (10). Indeed, a previous meta-analysis of over 15 thousand patients for a mean of 6.87 months after stroke reported an incidence of depressive disorder up to 33.5% (11). For early prevention of PSD, previous studies have attempted to identify the risk factors (e.g., female gender) (12–14). Nevertheless, the non-modifiable nature of the reported predictors has limited their clinical use.

Vitamin D is a fat-soluble vitamin vital for skeletal and extraskeletal health such as the maintenance of immune function, cancer prevention, and integrity of the cardiovascular system (15–18). Besides, vitamin D deficiency has been linked to an increased risk of postoperative delirium or depression (19, 20), suggesting its neuropsychiatric and neurocognitive function. Consistently, a growing body of evidence has shown a low circulating vitamin D level in a variety of populations with depressive disorder (21–23). Focusing on stroke survivors, several studies have also reported similar findings (24–26). As the incidence of low circulating vitamin D level was not uncommon in patients with stroke (24, 27, 28), it is of utmost importance to identify whether vitamin D level is a modifiable factor in the development of PSD. Nevertheless, pooled evidence through a systematic approach addressing the correlation between PSD and vitamin D level is scarce. Therefore, the primary outcome of the current meta-analysis aimed at investigating the association of PSD with the circulating vitamin D level, while the secondary outcomes included the relationships between PSD and other risk factors.

## 2. Methods

### 2.1. Protocol

The protocol of the current meta-analysis was registered on PROSPERO (registration no.: CRD42022381580). This study was reported based on the Preferred Reporting Items for Systematic Reviews and Meta-Analyses (PRISMA) statement.

### 2.2. Search strategy

We performed a comprehensive search of the Medline (ovid), EMBASE (ovid), Cochrane library, and Google Scholar databases from inception to December 2022 without placing restrictions on the publication year, language, or sample size. The following search terms were used: (”Stroke” or “CVA” or “Cerebrovascular Accident” or “Cerebrovascular Stroke*” or “Brain Infarction” or “Cerebral Infarction” or “Brain Stem Infarctions” or “Brain Ischemia” or “Brain infarction” or “Ischemic stroke” or “Stroke survivors”) and (”Depressi*” or “post-stroke depression” or “depressive symptoms”) and (”Vitamin D” or “Vitamin D deficienc*” or “vitamin D2” or “vitamin D3” or “25OHD” or “25(OH)D” or “25-hydroxyvitamin D” or “Hydroxycholecalciferols” or “hypovitaminosis D” OR “plasma/serum 25 (OH) Vitamin D”). Supplementary Table 1 summarizes the search strategy for one of these databases (i.e., Medline). Using the reference lists of relevant systematic reviews and those within the included articles, we manually searched for studies possibly missed on the initial search.

### 2.3. Eligibility criteria and study selection

The following criteria were used to determine if a peer-reviewed study was eligible for inclusion: (a) adults (i.e., 18 years of age or above) with stroke regardless of its mechanism (i.e., infarction or hemorrhage); (b) available information regarding serum vitamin D level before the diagnosis of PSD; and (c) randomized controlled studies or observational studies. The exclusion criteria were (1) studies that focused on patients with depression before stroke attack; (2) those in which details regarding vitamin D level or outcome were unavailable; (3) those presented as conference abstracts, reviews, letters, case reports; and (4) non-peer-reviewed articles.

Three steps were taken to determine study eligibility: (1) Duplicated records were removed by using the EndNote software; (2) Two authors independently screened the titles/abstracts of the retrieved records to identify articles for full-text reading; and (3) Studies were included if they fulfilled the inclusion criteria after full-text reading. All discrepancies in opinions were resolved through consultation with a third author.

### 2.4. Data extraction

Relevant information was retrieved from each study, including first author/publication year, characteristics of the study population (e.g., gender), number of patients, body mass index (BMI), comorbidities [e.g., hypertension, diabetes mellitus (DM)], National Institutes of Health stroke scale (NIHSS), circulating vitamin D levels, follow-up period, incidence of PSD, and country of publication. Two authors retrieved data independently using a specific data extraction sheet. Any disagreements were resolved via discussion. Whenever the relevant information was unclear or missing, we emailed the corresponding author for clarification.

### 2.5. Outcomes and definitions

The primary outcome of the current meta-analysis was the relationship between the risk of PSD and a low vitamin D status, which was defined based on individual studies. The secondary outcomes were the associations of other factors (e.g., hypertension) with the risk of PSD. The definition of PSD was in accordance with that of each study. Vitamin D deficiency was defined as a circulating 25[OH] D level < 50 nmol/L (27).

### 2.6. Risk of bias assessment and certainty of evidence

The quality of individual studies was scrutinized based on their risks of bias using the Newcastle-Ottawa Scale (NOS) that comprises eight items contained in three domains, namely selection, comparability, and outcome. A certain number of stars can be assigned to each item; while a maximum of two stars can be given to an item in the selection and outcome domains, each item in the comparability domain can receive a maximum of only one star. A study is regarded as “low-risk” if it has been assigned seven or more stars.

Two independent authors used the Grading of Recommendations Assessment, Development and Evaluation (GRADE) approach (29) for assessing the certainty of evidence for the study outcomes. Inconsistencies in opinions were settled through arbitration involving a third author.

### 2.7. Statistical analysis

All data were analyzed to calculate the pooled mean difference (MD) and odd ratio (OR) by using the random-effects model. The 95% confidence interval (CI) was also reported for each outcome. The heterogeneity was examined using *I*^2^ statistics with *I*^2^ values ≥50% representing substantial heterogeneity as previously reported (30, 31). To examine the reliability of the primary and secondary outcomes, sensitivity analysis was conducted using a leave-one-out approach. For an outcome shared by 10 or more studies, the existence of potential publication bias was discerned through visual inspection of a funnel plot. For primary outcome, meta-regression was conducted to identify the origin of heterogeneity by using the proportion of females and incidence of vitamin D deficiency as covariates. The Review Manager (RevMan) or comprehensive Meta-Analysis (CMA) V3 software (Biostat, Englewood, NJ, USA) were applied for statistical analyses. A probability value, *p*, less than 0.05 was deemed statistically significant.

## 3. Results

### 3.1. Literature search and characteristics of included studies

**FIGURE 1:**
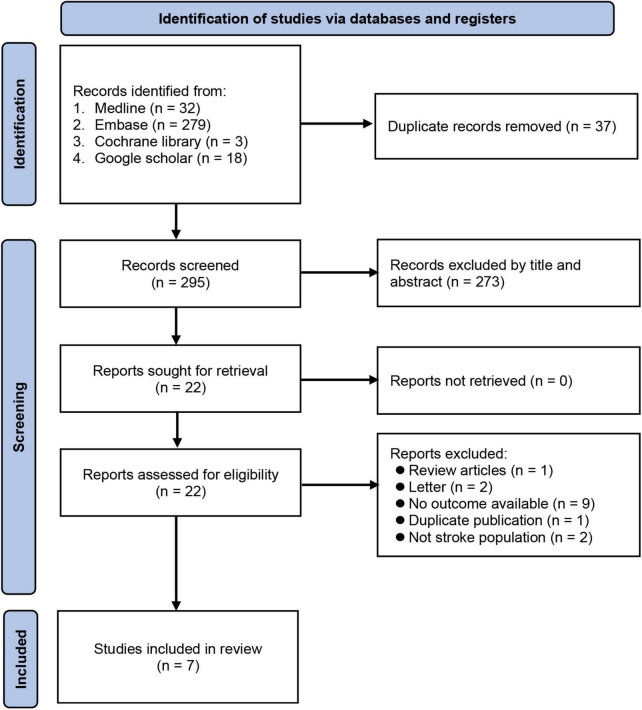
Flow chart for studies selection.

A total of 332 records were identified through literature search. Following a review of the titles and abstracts, 310 were excluded because of duplications or failure to meet the inclusion criteria. After a full-text review of the remaining 22 articles, 15 were further excluded for a variety of reasons shown in Figure 1. Finally, seven observational studies published between 2014 and 2022 were included in the current meta-analysis (24–26, 32–35).

**FIGURE 2:**
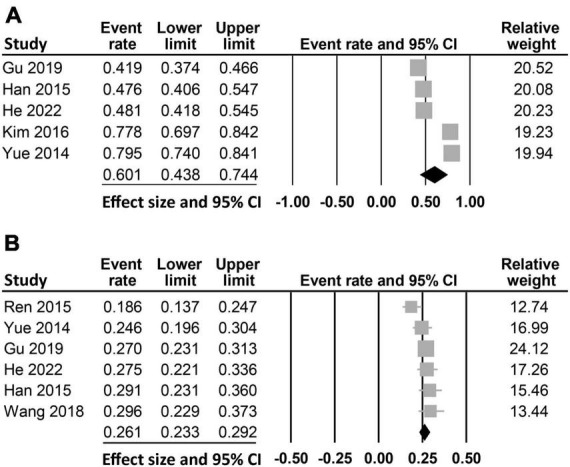
Pooled incidence of **(A)** vitamin D deficiency (pooled incidence: 60.1%, 95% CI: 43.8–74.4%, five studies) and **(B)** post-stroke depression (PSD) (pooled incidence: 26.1%, 95% CI: 23.3–29.2%, six studies). CI, confidence interval.

The characteristics of the 1,580 included patients diagnosed with stroke (mean or median age: 59.5–73.2 years; BMI: 23–25 kg/kg^2^) are summarized in Table 1. Six studies recruited mixed gender populations with the proportion of males ranging from 47.3 to 72.4%, while one study only focused on male patients (35). The number of patients were between 126 and 442. The diagnosis of PSD was based on DSM-IV criteria in three studies (26, 32, 33), Hamilton Depression Scale (HAMD) ≥ 7 in another three studies (24, 25, 35), and the Beck Depression Inventory II in one study (34). The follow-up time was one month in four studies (24, 25, 32, 33) and up to six months in another study (26), while two studies did not provide relevant information (34, 35). The incidence of vitamin D deficiency was available in five (24, 26, 32–34) out of the seven studies (range: 41.9–79.5%) with a pooled incidence of 60.1% (95% CI: 43.8–74.4%) (Figure 2A). The incidence of PSD was reported in six studies (range: 18.6–29.6%) (24–26, 32, 33, 35), while this information was not available in one study (34). Overall, the pooled incidence of PSD was 26.1% (95% CI: 23.3–29.2%) (Figure 2B). The studies were conducted in two countries including China (*n* = 6) (24–26, 32, 33, 35) and Korea (*n* = 1) (34). The quality of each study is shown in Table 1. All studies with a total number of stars ranging from 7 to 9 are regarded as “low-risk.”

**TABLE 1:** Characteristics of studies included (*n* = 7).

Studies	Population	Age (year)	Male (%)	BMI (kg/m^2^)	N	Incidence of vitamin d deficiency (definition 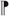 )	Diagnosis of depression	Follow-up (month)	Incidence of depression	Country	NOS
Gu et al. (32)	Patients with acute stroke	62.4	67.2	24	442	41.9% (<50 nmol/L)	DSM-IV criteria	1	27%	China	9
Han et al. (33)	Patients with acute stroke	62.6 vs. 61.6	47.3 vs. 72.4	24 vs. 25	189	47.6% (<50 nmol/L)	DSM-IV criteria	1	29.1%	China	8
He and Ruan (24)	Patients with acute stroke	63.3 vs. 63.2	53.1 vs. 65.7	24 vs. 24	233	48.1% (<50 nmol/L)	HAMD score ≥ 7	1	27.5%	China	9
Kim et al. (34)	Patients with stroke	62.1 vs. 65^†^	56.1 vs. 71.4^†^	NA	126	77.8% (<50 nmol/L)	Beck Depression Inventory II	NA	NA	Korea	8
Ren et al. (35)	Patients with acute stroke	61.7 vs. 59.5^‡^	100	24 vs. 23^‡^	194	NA	HAMD score ≥ 7	NA	18.6%	China	7
Wang et al. (25)	Patients with acute stroke	65 vs. 65	46.7 vs. 63.6	25 vs. 24	152	NA	HAMD score ≥ 7	1	29.6%	China	9
Yue et al. (26)	Patients with acute stroke	73.2 vs. 62.8	46.7 vs. 64.7	25 vs. 25	244	79.5% (<50 nmol/L)	DSM-IV criteria	6	24.6%	China	8

^†^Vitamin D deficiency vs. normal level group. ^‡^Non-smokers vs. smokers; 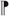
25[OH] D levels. NA, not available; HAMD, Hamilton Rating Scale for Depression; NOS, Newcastle-Ottawa Scale.

### 3.2. Outcomes

#### 3.2.1. Primary outcome

**FIGURE 3:**
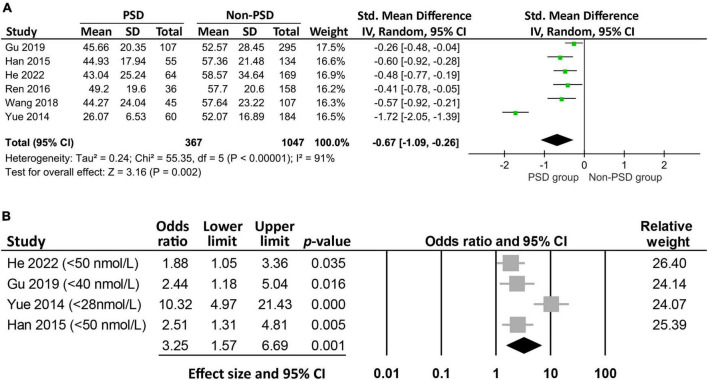
Forest plot comparing **(A)** the circulating vitamin D level between patients with and those without post-stroke depression (PSD), showing a lower circulating vitamin D level (mean difference: −13.94 nmol/L, 95% CI: −21.83 to −6.05, *p* = 0.0005, *I*^2^ = 91%, six studies, 1,414 patients) in patients with PSD; **(B)** the risk of PSD between patients with and those without low vitamin D levels, indicating a higher risk of PSD in patients with a lower level of vitamin D (odds ratio = 3.25, 95% CI: 1.57–6.69, *p* = 0.001, *I*^2^ = 78.7%, 1,108 patients). CI, confidence interval, SD, standard deviation; IV, inverse variance.

Six studies reported the circulating vitamin D level in patients with (*n* = 367) or without (*n* = 1,047) PSD (Figure 3A). Meta-analysis of these data revealed a lower vitamin D level in patients with PSD compared to those without (MD: −13.94 nmol/L, 95% CI: −21.83 to −6.05, *p* = 0.0005, *I*^2^ = 91%, six studies, 1,414 patients) (24–26, 32, 33, 35). Sensitivity analysis showed consistent findings, suggesting robustness of evidence. The circulating vitamin D levels were not available in one study (34), in which the authors reported a higher Beck depression Inventory II score in patients with vitamin D deficiency compared to that in those with a normal vitamin D level.

**FIGURE 4:**
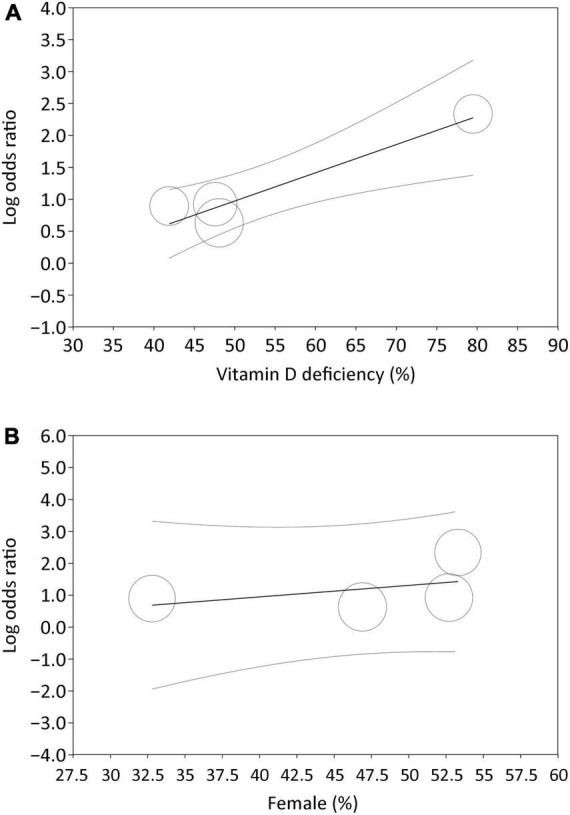
Meta-regression showing impact of **(A)** incidence of vitamin D deficiency (coefficient: 0.04, *p* = 0.0004), and **(B)** proportion of female gender (coefficient: 0.036, *p* = 0.467) on the association of low vitamin D status with risk of post-stroke depression, identifying incidence of vitamin D deficiency as a potential covariant affecting the relationship between a low vitamin D status and risk of post-stroke depression.

Analysis of the four studies that reported the event/total number or odds ratio on the relationship between low vitamin D status and PSD revealed a higher risk of PSD in patients with a lower circulating level of vitamin D (OR = 3.25, 95% CI:1.57–6.69, *p* = 0.001, *I*^2^ = 78.7%, 1,108 patients) (Figure 3B) (24, 26, 32, 33). The finding remained consistent on sensitivity analysis. Meta-regression revealed an association of the heterogeneity of this finding with the incidence of vitamin D deficiency (coefficient: 0.04, *p* = 0.0004) (Figure 4A), but not the proportion of female gender (coefficient: 0.036, *p* = 0.467) (Figure 4B).

#### 3.2.2. Secondary outcomes

**FIGURE 5:**
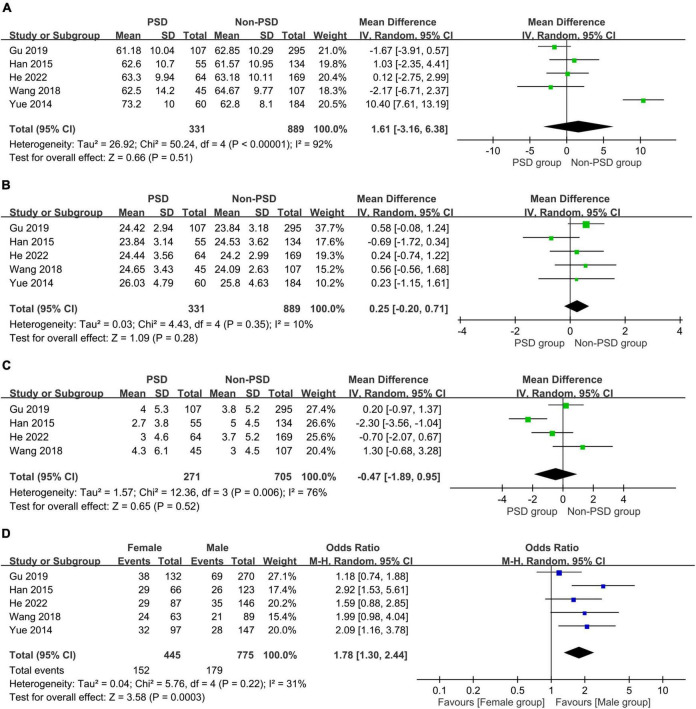
Forest plot showing the associations of post-stroke depression with **(A)** age (mean difference:1.61 years, 95% CI: −3.16 to 6.38, *p* = 0.51, *I*^2^ = 92%, five studies, 1,220 patients); **(B)** body mass index (BMI) (mean difference: 0.25 kg/m^2^, 95% CI: −0.2 to 0.71, *p* = 0.28, *I*^2^ = 10%, five studies, 1,220 patients); **(C)** education level (mean difference =−0.47, 96% CI: −1.89 to 0.95, *p* = 0.52, *I*^2^ = 76%, four studies, 976 patients); and **(D)** female gender (odds ratio = 1.78, 95% CI: 1.3–2.44, *p* = 0.003, *I*^2^ = 31%, five studies, 1,220 patients), indicating an correlation of female gender with the risk of post-stroke depression. MH, Mantel-Haenszel; IV, inverse variance; CI, confidence interval.

The associations of age, female gender, BMI, and level of education with PSD are summarized in Figure 5. There were no differences in age (MD:1.61 years, 95% CI: −3.16 to 6.38, *p* = 0.51, *I*^2^ = 92%, five studies, 1,220 patients) (Figure 5A) (24–26, 32, 33), BMI (MD: 0.25 kg/m^2^, 95% CI: −0.2 to 0.71, *p* = 0.28, *I*^2^ = 10%, five studies, 1,220 patients) (Figure 5B) (24–26, 32, 33), and level of education (MD =−0.47, 96% CI: −1.89 to 0.95, *p* = 0.52, *I*^2^ = 76%, four studies, 976 patients) (Figure 5C) (24, 25, 32, 33) in patients with or without PSD. However, women showed a higher risk of PSD compared to that in men (OR = 1.78, 95% CI: 1.3–2.44, *p* = 0.003, *I*^2^ = 31%, five studies, 1,220 patients) (Figure 5D) (24–26, 32, 33).

**FIGURE 6:**
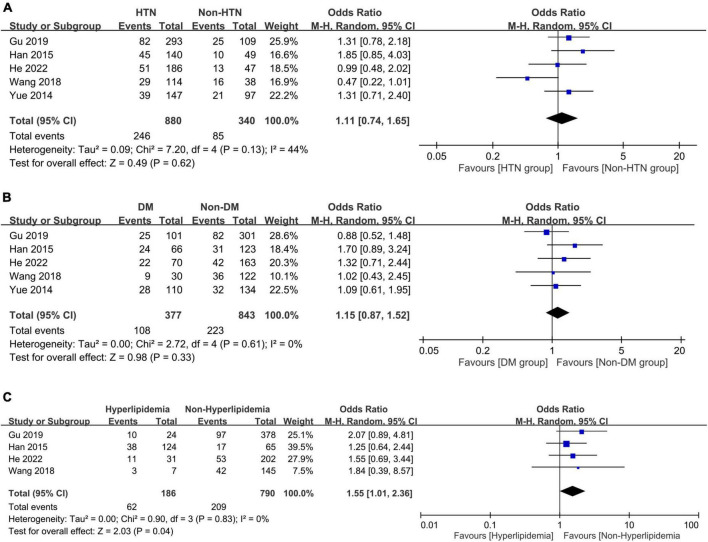
Forest plot showing the associations of post-stroke depression with **(A)** hypertension (HTN) (odds ratio = 1.11, 95% CI: 0.74–1.65, *p* = 0.62, *I*^2^ = 44%, five studies, 1,220 patients); **(B)** diabetes mellitus (DM) (odds ratio = 1.15, 95% CI: 0.87–1.52, *p* = 0.33, *I*^2^ = 0%, five studies, 1,220 patients); and **(C)** hyperlipidemia (odds ratio = 1.55, 95% CI: 1.01–2.36, *p* = 0.04, *I*^2^ = 0%, four studies, 976 patients), demonstrating a link between hyperlipidemia and the risk of post-stroke depression. CI, confidence interval; MH, Mantel-Haenszel; CI, confidence interval.

**FIGURE 7:**
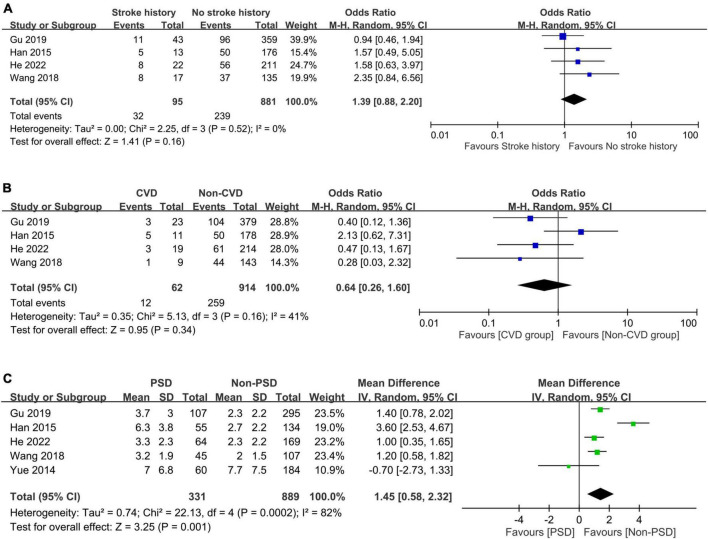
Forest plot showing the association of post-stroke depression (PSD) with **(A)** stroke history (odds ratio = 1.39, 95% CI: 0.88–2.2, *p* = 0.16, *I*^2^ = 0%, four studies, 976 patients); **(B)** cardiovascular disease (CVD) (odds ratio = 0.64, 95% CI: 0.26–1.6, *p* = 0.34, *I*^2^ = 41%, four studies, 976 patients); and **(C)** score on the National Institute of Health stroke scale (NIHSS) (mean difference: 1.45, 95% CI: 0.58–2.32, *p* = 0.001, *I*^2^ = 82%, five studies, 1,220 patients), revealing a correlation between NIHSS score and the occurrence of post-stroke depression. CI, confidence interval; MH, Mantel-Haenszel.

The associations of PSD with comorbidities including systemic and vascular diseases are shown in Figures 6, 7, respectively. The presence of hypertension (OR = 1.11, 95% CI: 0.74–1.65, *p* = 0.62, *I*^2^ = 44%, five studies, 1,220 patients) (Figure 6A) (24–26, 32, 33) or diabetes mellitus (OR = 1.15, 95% CI: 0.87–1.52, *p* = 0.33, *I*^2^ = 0%, five studies, 1,220 patients) (Figure 6B) (24–26, 32, 33) did not correlate with a higher risk of PSD, while patients with hyperlipidemia were at risk of PSD compared to those without (OR = 1.55, 95% CI: 1.01–2.36, *p* = 0.04, *I*^2^ = 0%, four studies, 976 patients) (Figure 6C) (24, 25, 32, 33). There was no increased risk of PSD in patients with stroke history (OR = 1.39, 95% CI: 0.88–2.2, *p* = 0.16, *I*^2^ = 0%, four studies, 976 patients) (Figure 7A) (24, 25, 32, 33) or cardiovascular disease (OR = 0.64, 95% CI: 0.26–1.6, *p* = 0.34, *I*^2^ = 41%, four studies, 976 patients) (Figure 7B) (24, 25, 32, 33). However, patients with PSD had a higher NIHSS score at stroke onset than that in those without PSD (MD: 1.45, 95% CI: 0.58–2.32, *p* = 0.001, *I*^2^ = 82%, five studies, 1,220 patients) (Figure 7C) (24–26, 32, 33).

### 3.3. Certainty of evidence

The certainty of evidence is demonstrated in Supplementary Table 2. For the primary outcome of the associations of circulating vitamin D concentration with the occurrence and risk of PSD, the certainty of evidence was very low. Regarding the secondary outcomes of the impacts of other factors on the occurrence and risk of PSD, the certainty of evidence was low for BMI, female gender, hypertension, DM, stroke history. On the other hand, the certainty of evidence was very low for age, level of education, hyperlipidemia, cardiovascular disease, and NIHSS scores.

## 4. Discussion

Despite reports from observational studies of a connection between a low circulating vitamin D level and PSD (24–26) that affects one-third of patients following acute stroke (14), there was no pooled evidence showing their correlation or identifying the influences of other potential confounders. Through focusing on patients with stroke, the current meta-analysis demonstrated that the pooled incidences of vitamin D deficiency and PSD were 60.1 and 26.1%, respectively. Stroke survivors with PSD had a lower circulating vitamin D concentration compared to those without (MD: −13.94 nmol/L). Consistently, a lower level of circulating vitamin D was associated with a three-fold increase in the risk of PSD (OR: 3.25). In addition, examining the relationship between ten other variables and PSD identified three potential predictors including female gender, hyperlipidemia, and a high NIHSS score. There were no associations of PSD with age, BMI, level of education, hypertension, DM, stroke history, and cardiovascular disease (all *p* > 0.05).

Although previous studies have linked a low vitamin D level to an increased risk of depression (36–38), the exact mechanisms underlying the correlation are not fully understood (39, 40). One possibility is that vitamin D plays a role in the synthesis of neurotransmitters (e.g., serotonin, and norepinephrine, dopamine) associated with mood regulation (40). Vitamin D may also affect the immune system and inflammation, which have been reported to contribute to the development of depression (41, 42). A previous review suggested that a low vitamin D level may disturb the HPA axis (43), thereby causing a dysregulation of stress and mood. This finding was further supported by the result of a previous experimental study that demonstrated antidepressant-like effect of vitamin D that acts through the brain-derived neurotrophic factor (BDNF) and NT-3/NT-4 signaling pathways in the hippocampus (44). In addition, gender may have a role to play in the development of PSD. A prior animal study demonstrated significant improvement in the depressive behaviors of PSD rats through estrogen administration, possibly involving tyrosine kinase B (TrkB)/brain-derived neurotrophic factor (BDNF)/cAMP response element-binding protein (CREB) signaling in the hippocampus (45, 46). Consistent finding was noted in a cross-sectional multi-ethnic cohort clinical investigation including 3,017 men and 2,929 women that reported an association between a low vitamin D level and a lower estradiol level in women (47). Besides, a large cohort study involving 139,128 middle-aged adults found an association of low vitamin D levels with the development of new-onset depression as well as sustained depressive symptoms (48), supporting the role of vitamin D in the psychiatric disorder. Despite the inconsistent findings from two previous meta-analytic studies (49), evidence from several meta-analyses focusing on non-stroke individuals still reported a potential relationship between the development of depression and low serum vitamin D concentrations (22, 23, 50).

The variation in the prevalence of PSD between 18 and 33% among individual studies (4) may be attributed to differences in study designs and methods of depression evaluation (51). Despite advances in diagnosis and treatment of PSD, our finding of a pooled incidence of PSD of 26.1% from six studies published between 2014 and 2022 was comparable to that of a previous large-scale meta-analysis of 51 studies conducted between 1977 and 2002 that reported a 33% pooled prevalence of the condition (51). Therefore, the current study suggests that the development of PSD was not effectively prevented in recent decades. PSD has been shown to be associated with poor short- and long-term outcomes, including a prolonged hospital stay, profound cognitive deficits, serious functional disability, a poor life quality, impaired rehabilitation outcomes, and even mortality (4, 52–57). The urgency of the need for PSD prophylaxis has highlighted the utmost importance of identifying the risk factors for PSD. The well-known predictors of PSD include female gender, stroke severity, physical disability, cognitive impairment, depression before stroke, as well as a lack of family and social support (12–14). In the current meta-analysis, we also found that female gender and stroke severity measured with NIHSS were both predictors of PSD. Despite these findings, most risk factors were non-modifiable, underscoring the difficulty in PSD prophylaxis. Although a low circulating vitamin D has been reported to be a risk factor for depression (21–23), our report is the first meta-analysis addressing this modifiable factor in the post-stroke setting.

According to a large-scale study involving 14,302 adults aged from 18 to 65 years, the prevalence of vitamin D insufficiency and deficiency were 32.7 and 41.9%, respectively (58). One of the interesting findings of that study was a lower circulating vitamin D level in women than in men (58), which may partially explain an elevated risk of PSD associated with the female gender in the current study. With regard to the effect of age, a previous study of elderly patients in a rehabilitation unit revealed a prevalence of vitamin D deficiency up to 44% (59). Focusing on disease status, previous studies have also shown an equally high prevalence of vitamin D deficiency in patients with stroke, ranging from 30 to 62.9% (24, 27, 28). The current meta-analysis revealed a pooled incidence of 60.1% of vitamin D deficiency, which was consistent with that reported in current literature. Therefore, our findings and those from previous studies suggest a ubiquity of a low circulating vitamin D level in both the general and elderly population, especially in those with stroke (58, 59), for whom a routine assessment of circulating vitamin D levels may be necessary.

Current pharmacological and non-pharmacological therapies for PSD include cognitive behavior therapy (60), transcranial magnetic stimulation (61), hyperbaric oxygen therapy (62), selective serotonin reuptake inhibitors (SSRIs) (63, 64), and acupuncture combined with antidepressants (65). Although SSRIs are recommended as the first-line treatment, their effectiveness and tolerability remain controversial (64). A previous meta-analysis of 10 trials including a total of 5,370 patients supported the efficacy of early SSRI therapy for PSD prophylaxis despite a lack of improvement in the patient’s functional independence (63). However, adverse side effects from SSRIs (e.g., seizure and nausea) are another clinical concern (63). On the other hand, although previous meta-analyses reported the beneficial effects of non-pharmacological approaches including cognitive behavior therapy (23 studies with 1,972 patients), hyperbaric oxygen therapy (27 randomized controlled trials with 2,250 participants), and transcranial magnetic stimulation (7 randomized controlled trials with 351 participants) against the depressive symptoms of PSD (60–62), the pooled evidence remained inconclusive because of the limitations of the included studies (e.g., poor methodological quality or limited number of patients). In respect of the impact of vitamin D supplementation on symptoms of depression, the findings of previous studies in the non-stroke population remained inconsistent (66–68) and there were no relevant studies focusing on PSD. Nevertheless, there was evidence showing an association of vitamin D supplementation with better rehabilitation outcomes in patients diagnosed with stroke (69). Accordingly, this approach may still be recommended for this patient population despite the lack of evidence supporting the beneficial effects of vitamin D supplementation on PSD.

Our finding of an association of a low vitamin D status with PSD may partly be explained by the link between the former and poor sleep quality (70). A previous retrospective study on 1,619 individuals diagnosed with acute ischemic stroke reported an increased risk of subsequent depression and anxiety in those with persistent poor baseline sleep quality (71). Another study further supported the correlation by showing an improved sleep quality in patients with chronic pain by using vitamin D supplements (72), underscoring the possibility that vitamin D-related impairment of sleep quality may contribute to the risk of PSD. Further clinical investigations are needed to address this issue.

Despite our provision of pooled evidence on the relationship between PSD and circulating vitamin D level through analyzing multiple studies, there were several limitations in the current meta-analysis. First, previous investigations have suggested that certain factors, including racial/ethnic differences, may have an impact on the risk of PSD (73–75). As all of the included studies were conducted in Asian countries, our findings may not be applicable to populations of other ethnic backgrounds. Besides, the possibility of publication bias may exist. Second, the impacts of other confounding factors such as socioeconomic status (76), obesity (77), and nutrient deficiency/diet (78, 79), which were not evaluated in the current study, may bias our results. Third, our finding should be interpreted with caution because meta-analysis of observational studies is unable to establish a causal relationship. Fourth, there was a high heterogeneity in our primary outcome probably attributable to the variations in follow-up time and the diagnostic approach to depression. In fact, because the diagnosis of depression (e.g., DSM-IV criteria, and HAMD Score) was based on self-reported assessment in the included studies, the possibility of reporting bias could not be ruled out. Fifth, we were unable to investigate a dose-response relationship between the risk of PSD and circulating vitamin D concentration due to a lack of relevant data. Sixth, despite the previous identification of a low circulating vitamin D level as a risk factor for stroke (80), no data on serum vitamin D concentration before stroke occurrence were available in the included studies so that the relationship between vitamin D level, stroke, and depression could not be fully elucidated. Finally, the impacts of low vitamin D levels on other prognostic outcomes in this population were not assessed. Further studies are needed to address these issues.

## 5. Conclusion

The results of the current meta-analysis suggested an association of a low circulating vitamin D level with an over three-fold increase in the risk of post-stroke depression. Moreover, female gender, hyperlipidemia, high NIHSS score were linked to an increased risk or occurrence of post-stroke depression. Our findings may indicate the need for routine screening of circulating vitamin D concentration in patients with stroke and in other high-risk populations. Taking into consideration the limited number of studies available and their observational nature, further well-controlled prospective investigations are warranted to verify the correlation between vitamin D level and the development of post-stroke depression as well as to explore the efficacy of potential intervention strategies (e.g., vitamin D supplementation).

## Data availability statement

The original contributions presented in this study are included in the article/Supplementary material, further inquiries can be directed to the corresponding authors.

## Author contributions

K-CH and J-YW: conceptualization. AI: methodology and software. C-CC and Y-JC: validation. K-CH and S-WL: formal analysis. K-FW and I-WC: investigation. I-WC: resources. I-WC and K-CH: data curation. K-CH, J-YW, I-WC, and C-KS: writing—original draft preparation. K-CH, I-WC, and C-KS: writing—review and editing. C-KS: visualization and supervision. All authors have read and agreed to the published version of the manuscript.
