# Supplementary material for: Association of a low vitamin D status with risk of post-stroke depression: A meta-analysis and systematic review
Source: Front Nutr. 2023 Feb 16;10:1142035. doi: 10.3389/fnut.2023.1142035 (PMC9983120; doi:10.3389/fnut.2023.1142035)
Supplement: Supplementary file 1 [file Data_Sheet_1.docx]

**Supplemental Table 1.** Search strategies

| Database |  | Search syntax |
| --- | --- | --- |
| **MEDLINE (Ovid)** | #1 | ("Stroke" or "CVA" or "Cerebrovascular Accident" or "Cerebrovascular Stroke*" or "Brain Infarction" or "Cerebral Infarction" or "Brain Stem Infarctions" or "Brain Ischemia" or "Brain infarction" OR "Ischemic stroke" OR "stroke survivors").mp. |
|  | #2 | exp "Stroke"/ or exp "Brain Infarction"/ |
|  | #3 | ("Depressi*" or "post-stroke depression" or "depressive symptoms").mp. |
|  | #4 | exp "Depression"/ |
|  | #5 | ("Vitamin D" or "Vitamin D Deficienc*" or "vitamin D2" or "vitamin D3" or "25OHD" or "25(OH)D" or "25-hydroxyvitamin D" or "Hydroxycholecalciferols" or "hypovitaminosis D" OR "plasma/serum 25 (OH) Vitamin D").mp. |
|  | #6 | exp "Vitamin D"/ or exp "Vitamin D Deficiency"/ |
|  | #7 | (1 or 2) and (3 or 4) and (5 or 6) |

| **Supplemental table 2.** Summary of findings for the main comparison | | | | | | |
| --- | --- | --- | --- | --- | --- | --- |
| Outcomes | Effect (Risk or mean) | | Relative effect (95% CI) | № of participants  (studies) | Certainty of the evidence (GRADE) | Comments |
|  | Intervention group | Control group |  |  |  |  |
| Vitamin D level with or without PSD | - | - | MD: -13.94  (-21.83 to -6.05) | 1414 (6 studies) | ⨁◯◯◯ Very Low | a, b |
| Risk of PSD with low level of vitamin D | - | - | OR: 3.25  (1.57 to 6.69) | 1108  (4 studies) | ⨁◯◯◯ Very Low | a, b |
| Age | - | - | MD 1.61 (-3.16 to 6.38) | 1220 (5 studies) | ⨁◯◯◯ Very Low | b |
| Body mass index | - | - | MD 0.25 (-0.2 to 0.71) | 1220 (5 studies) | ⨁⨁◯◯ Low | - |
| Female | 152/445 | 179/775 | RR 1.78 (1.3 to 2.44) | 1220 (5 studies) | ⨁⨁◯◯ Low | - |
| Level of education | - | - | MD -0.47 (-1.89 to 0.95) | 1220 (5 studies) | ⨁◯◯◯ Very Low | b |
| Hypertension | - | - | OR 1.11 (0.74 to 1.65) | 1220 (5 studies) | ⨁⨁◯◯ Low | - |
| Diabetes mellitus | - | - | OR 1.15 (0.87 to 1.52) | 1220  (5 studies) | ⨁⨁◯◯ Low | - |
| Hyperlipidemia | 62/186 | 209/790 | RR 1.55  (1.01 to 2.36) | 976  (4 studies) | ⨁◯◯◯ Very Low | a |
| Stroke history | 32/95 | 239/881 | RR 1.39  (0.88 to 2.2) | 976  (4 studies) | ⨁⨁◯◯ Low | - |
| Cardiovascular disease | 12/62 | 259/914 | RR 0.64  (0.26 to 1.6) | 976  (4 studies) | ⨁◯◯◯ Very Low | a |
| NIHSS score | - | - | MD 1.45  (0.58 to 2.32) | 1220  (5 studies) | ⨁◯◯◯ Very Low | b |

PSD: post-stroke depression; NIHSS: National Institutes of Health Stroke Scale

Comments:

^a^wide 95% CI; ^b^The I square is more than 50%.

GRADE Working Group grades of evidence:
High certainty: We are very confident that the true effect lies close to that of the estimate of the effect
Moderate certainty: We are moderately confident in the effect estimate: The true effect is likely to be close to the estimate of the effect, but there is a possibility that it is substantially different
Low certainty: Our confidence in the effect estimate is limited: The true effect may be substantially different from the estimate of the effect
Very low certainty: We have very little confidence in the effect estimate: The true effect is likely to be substantially different from the estimate of effect
